# Experimental Periodontitis Deteriorated Atherosclerosis Associated With Trimethylamine N-Oxide Metabolism in Mice

**DOI:** 10.3389/fcimb.2021.820535

**Published:** 2022-01-18

**Authors:** Lingling Xiao, Lingyan Huang, Xin Zhou, Dan Zhao, Yan Wang, Haiyan Min, Shiyu Song, Weibin Sun, Qian Gao, Qingang Hu, Sijing Xie

**Affiliations:** ^1^Nanjing Stomatological Hospital, Medical School of Nanjing University, Nanjing, China; ^2^Center for Translational Medicine and Jiangsu Key Laboratory of Molecular Medicine, Medical School of Nanjing University, Nanjing, China; ^3^Department of Stomatology, The Second People’s Hospital of Taizhou, Taizhou, China; ^4^The Affiliated Stomatological Hospital of Soochow University, Suzhou, China; ^5^The Second Affiliated Hospital of Nanjing University of Chinese Medicine, Nanjing, China

**Keywords:** trimethylamine N-oxide, periodontitis, *Porphyromonas gingivalis*, gut microbiota, flavin-containing monooxygenase 3

## Abstract

**Background:**

Periodontitis is considered a risk factor for atherosclerosis, but the mechanism is not clear. It was reported that oral administration of *Porphyromonas gingivalis* altered the gut microbiota in mice. Gut dysbiosis and the intestinal metabolite trimethylamine N-oxide (TMAO) were verified to be associated with atherosclerosis. Therefore, the possible TMAO-related mechanism between periodontitis and atherosclerosis needs to be explored.

**Methods:**

Experimental periodontitis was established by oral administration of *P. gingivalis* for 2 months in ApoE^−/−^ mice. Mouse hemi-mandibles were scanned using Micro-CT. Quantification of TMAO was performed using liquid chromatography–tandem mass spectrometry. Mouse feces were collected and the bacterial DNA was extracted, then the gut microbiota was analyzed using 16S rRNA genes. Atherosclerotic lesion areas were quantified. Livers, small intestines, and large intestines were analyzed for gene expression.

**Results:**

Aggravated atherosclerosis plaques were found in experimental periodontitis mice. Plasma TMAO, a pathogenic factor of atherosclerosis, was initially found to be increased in periodontitis mice. Changes in the composition and abundance of the intestinal microflora of periodontitis mice were found. Flavin monooxygenase 3 (FMO3), the catalyzing enzyme of TMAO in the liver, was significantly increased, accompanied by an increase of IL-6 in liver, the abnormal intestinal integrity and enhanced plasma LPS. The IL-6 and LPS were verified to be able to increase FMO3 in HepG2 cells.

**Conclusion:**

Our research discovered that experimental periodontitis in ApoE^−/−^ mice induced gut dysbiosis and an increase in TMAO. These results suggest a possible mechanism by which periodontitis may accelerate atherosclerosis by influencing the intestinal microbes and the metabolism, which were triggered by inflammation of the liver and intestine.

## Introduction

Periodontitis is a chronic inflammatory disease of periodontal tissues caused by dental biofilm and calculus (Pihlstrom et al., 2005). Atherosclerosis (AS) is a slowly progressive disease characterized by lipid accumulation in the outer arterial tunica intima (Xu et al., 1990). Epidemiological and biological studies indicate that periodontitis may be an important risk factor for cardiovascular disease and atherosclerosis (Socransky and Haffajee, 2005; Kebschull et al., 2010; Olivier et al., 2011). So far there are two main viewpoints about the mechanism of periodontitis related atherosclerosis. One suggested that the local inflammatory nidus in periodontal pocket could induce atherosclerosis by instigating the inflammatory cascade (Hegde and Awan, 2019). The second one proposed that an invasion of the periodontal bacteria through diseased pockets into bloodstream for atherosclerosis induction (D’Aiuto et al., 2005; Leticia et al., 2013; Kholy et al., 2015). However, the mechanism of how exactly periodontitis induces the atherosclerosis is still unclear.

Trimethylamine N-oxide, a metabolite of gut flora, has shown promise as a special indicator of atherosclerosis (Koeth et al., 2013). The circulatory TMAO may be associated with cardiovascular risks by changing enterohepatic cholesterol and bile acid metabolism that control the pathway required to eliminate cholesterol from the body (Tang and Hazen, 2014; Chistiakov et al., 2015; Wang and Zhao, 2018). Wang et al. found that TMAO promoted upregulation of multiple macrophage scavenger receptors linked to atherosclerosis and supplementation with TMAO promoted atherosclerosis in ApoE^−/−^ mice (Wang et al., 2011). TMAO is converted from trimethylamine (TMA) in the liver by the oxidative effect of flavin monooxygenases (FMOs) (Schugar and Brown, 2015). TMA is a small molecule produced by the gut bacteria metabolizing some components in foods, such as choline, phosphatidylcholine, and carnitine (Wang and Zhao, 2018).

The intestine is the largest microbial habitat in the human body, holding over 1,000 microbial phylotypes, and the number is up to 100 trillion, which is 100 times the human genome (Ley et al., 2006; Rajilićstojanović et al., 2010). In the last decade, there have been studies demonstrating that gut dysbiosis is associated with atherosclerosis (Tang et al., 2017). Compared with normal controls, the plaque areas of germ-free ApoE^−/−^ mice were significantly bigger after the same diet for 3–4 months (Stepankova et al., 2010), suggesting that the normal gut microbiota was protective in the development of atherosclerosis. Moreover, mice that were gavaged with cecal microbial contents from atherosclerosis-prone or atherosclerosis-resistant mice, exhibited similar symptoms with their donor mice (Gregory et al., 2015). Strikingly, a clinical study stratified the human gut microbiota into three enterotypes characterized by *Bacteroides*, *Prevotella*, and *Ruminococcus*, respectively, and the atherosclerosis patients were overrepresented in enterotype 3 represented by *Ruminococcus* (Karlsson et al., 2012). Further studies performed by a Japanese research group reported that oral administration of *Porphyromonas gingivalis* (*P. gingivalis*), a periodontal pathogen, could alter the gut microbiota and induce systemic inflammation (Arimatsu et al., 2014; Nakajima et al., 2015), thus linking the key periodontal pathogen with the gut microbiota and systemic inflammation.

Therefore, we hypothesized that periodontitis may induce gut dysbiosis and abnormal hepato-intestinal metabolism, leading to the accelerated development of atherosclerosis. In this study, *P. gingivalis* was used to generate an experimental periodontitis model in ApoE^−/−^ mice. Aggravated atherosclerosis plaques, enhancement of TMAO in peripheral blood and increased FMO3, the catalyzing enzyme of TMAO in the liver, were observed in the experimental periodontitis mice, compared with the control. It may hint at a new pathway for periodontitis to promote atherosclerosis: the alterations of intestinal microbes and their metabolites originated by periodontitis.

## Materials and Methods

### Mice

Ten 8-week-old male ApoE^−/−^ mice, and ten 8-week-old male C57BL/6J mice were obtained from the Model Animal Research Center of Nanjing University (Nanjing, China). The mice were housed in a controlled pathogen-free environment with free access to food and water for acclimatization (Cani et al., 2008), and then randomized to two groups: the control group and the experimental periodontitis group. They were fed Western Diet (WD) consisting of 21% (w/w) fat, 0.2% cholesterol, and 0% choline (TD88137–Harlan) for an additional 8 weeks. All experimental procedures were reviewed and approved by the Institutional Animal Care and Use Committee of Nanjing University (IACUC-D2102033).

### Bacterial Culture and Oral Administration

*P. gingivalis* strain 33277 was cultured in a Brain Heart Infusion Broth (Beyotime Biotechnology, China) in an anaerobic environment for 48 h at 37 °C. The anaerobic state was kept by AnaeroPack (Mitsubishi Gas Chemical Company, Inc, Japan) in an anaerobic jar (Oxoid, England) (Arimatsu et al., 2014). The concentration of bacteria was determined with a spectrophotometer (SpectraMax M3, Molecular Devices, USA) at an optical density of 600 nm (OD = 10^9^ P*. gingivalis* per ml) (Zhang et al., 2014). A total of 10^9^ CFU of live *P. gingivalis* was collected by centrifugation, and then resuspended in 100 μl Phosphate Buffered Saline (PBS) with 2% carboxymethyl cellulose (Sigma-Aldrich, America). The bacterial suspension was given to each of the five mice gingival margin of the molars 5 times a week for 8 weeks. The other five mice in the control group were sham-administrated without the *P. gingivalis*. Twenty-four hours after the final intervention, the feces were collected from the live mice, and then the mice were anesthetized for tissues collection (Arimatsu et al., 2014).

### Quantification of Mandibular Alveolar Bone Resorption

The hemi-mandibles were scanned using a high-resolution Micro-CT (SkyScan1176, Bruker, Germany) for alveolar bone loss evaluation. After scanning, a set of slices were used for three-dimensional reconstructions. All images were reoriented such that the cement-enamel junction (CEJ) and the alveolar bone crest (ABC) appeared in the Micro-CT slice that was to be analyzed. The alveolar bone loss was measured from the CEJ to the ABC of the mesial surface of the first molar on the sagittal plane (Park et al., 2007; Madeira et al., 2013).

### Quantification/Histology of Atherosclerotic Lesion Area and Liver

The collected mouse hearts were embedded in tissue freezing medium (Sakura, America), and then sliced and stained with oil red O. The stained plaque area of the aortic sinus was analyzed using Image J 1.37c (National Institutes of Health, USA) (Tomoki et al., 2011). Hematoxylin–eosin-stained (H&E) sections of the aortic arch and liver were used for morphometric analysis (Kesavalu et al., 2012).

### Quantification of Plasma TMAO, TMA, Choline, Creatinine, Betaine and L-Carnitine

Quantification of TMAO, TMA, Choline, Creatinine, Betaine, and L-Carnitine was performed using stable isotope dilution ultra-high-performance liquid chromatography–tandem mass spectrometry (UHPLC–MS/MS). The analysis used a Waters ACQUITY UPLC HSS T3 column (2.1 ∗ 100 mm, 1.8 um). Mobile phases A and B, respectively, comprised 0.1% formic acid and 0.1% acetonitrile in water. MS quadrupole and ion source temperatures were separately taken as 100 and 650°C. The ion transitions were m/z 76.1 → 58.1 for TMAO, m/z 60 → 40.5 for TMA, m/z 118.1 → 58.1 for Betaine, m/z 104.1 → 45 for Choline, m/z 114.1 → 44 for Creatinine and m/z 162.1 → 85 for L-Carnitine.

### Lipoprotein and Serum Analysis

The mouse serum samples were collected by extracting the eyeballs, and assayed for total cholesterol (TC), triglyceride (TG), low-density lipoprotein (LDL), high-density lipoprotein (HDL), oxidized low-density lipoprotein (ox-LDL), lipopolysaccharide (LPS), interleukin (IL)-6, interleukin (IL)-1β, and tumor necrosis factor (TNF)-α. The levels of ox-LDL, LPS, IL-6, IL-1β, and TNF-α were determined by ELISA (Dakewe, China). Enzymatic colorimetry (Biosino Bio-Technology & Science Inc, China) was used for serum TC and TG analysis. The LDL and HDL concentrations were detected by the LDL/HDL-Cholesterol Kit (Biosino Bio-Technology & Science Inc, China) (Lalla et al., 2003).

### Cell Culture

The HepG2 cell line was obtained from ATCC. HepG2 cells were cultured in DMEM high glucose medium (10% fetal bovine serum, 1% penicillin and streptomycin) at 37°C and 5% CO_2_ saturated humidity. When the cell adherence rate reached 80 to 90%, the cells were digested with trypsin, and the cell viability was above 90% for subpassage. One day before the experiment, the cells were digested with 0.25% trypsin and inoculated with DMEM high glucose medium containing 10% fetal bovine serum into a 6-well culture plate. The agents were added after the cells were completely adhered to the wall. There were 5 wells in each group.

### Analysis of Gene Expression in the Samples

The total RNA was extracted from the mouse liver, abdominal adipose, large intestine, small intestine samples, and HepG2 cells by RNAsimple Total RNA Kit (Tiangen, China). Reverse transcription PCR was carried out in the PCR Thermal Cyclers (Applied Biosystems, Thermo Fisher, America) for cDNA synthesis with PrimeScript™ RT reagent Kit with gDNA Eraser (Takara, Japan). Primers for real-time PCR were purchased from GenScript, and the primer sequences were listed in **Supplemental Table 1**. The gene expression analysis was realized finally in a final volume of 20 μl consisted by 0.5–5 ng cDNA, 900 nM each of the forward and reverse primers, and 10 μl iTaq Universal SYBR Green Supermix (Bio-Rad, America) in QuantStudio 7 Flex Real-Time PCR System (Applied Biosystems, Thermo Fisher, America). The PCR conditions were 2 min at 50°C, 10 min at 95°C followed by 40 cycles of two-step PCR denaturation at 95°C for 15 s and annealing extension at 60°C for 60 s. The relative amount of each studied mRNA was normalized to the relative quantity of glyceraldehyde-3-phosphate dehydrogenase (GAPDH) mRNA, and the data were analyzed according to the 2^−△△CT^ method (Cani et al., 2008; Nakajima et al., 2015).

### DNA Extraction From Samples and Gut Microbiota Genome Sequencing

The mouse feces were collected after *P. gingivalis* administration for two months, and the bacterial DNA was extracted from feces by TIANamp Stool DNA Kit (Tiangen, China). The bacterial DNA extracted from feces was collected for gut microbiota analysis. The V3–V4 region of the 16S rRNA genes was amplified by PCR using a primer set (F: CCTAYGGGRBGCASCAG, R: GGACTACNNGGGTATCTAAT). The qualified DNA was used for sequencing analysis in Illumina HiSeq platform at the Novogene Bioinformatics Institute (Beijing, China) (Ma et al., 2018).

### Statistical Analysis

The statistical analysis of phenotypic traits was performed using SPSS22. For statistical analysis, either a student t-test or one-way ANOVA was performed, and the significance level was set at P = 0.05 which was shown in the figure legends.

## Results

### Experimental Periodontitis Caused Deteriorated Atherosclerosis and Increased TMAO in the Peripheral Blood of the ApoE^−/−^ Mice

**Figure 1:**
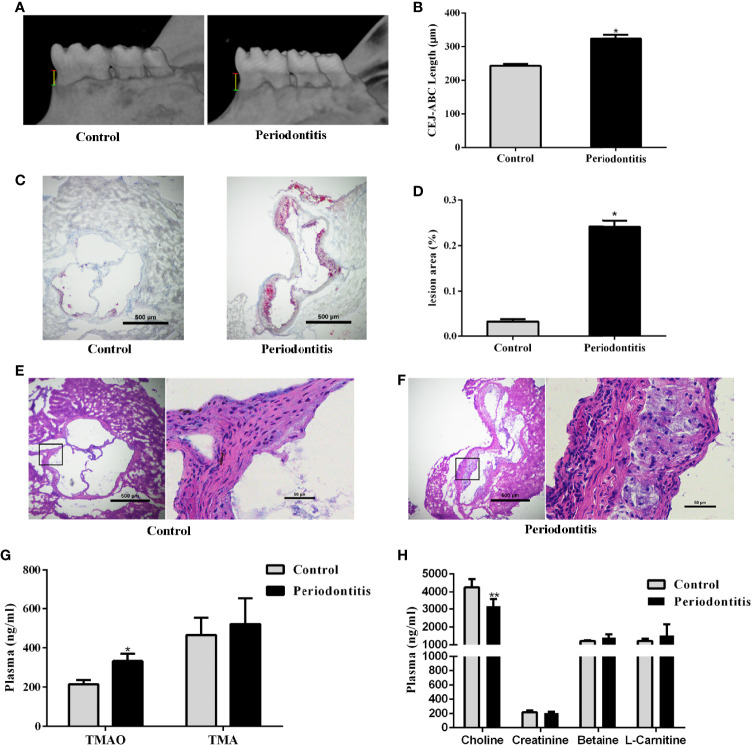
Effect of periodontitis on alveolar bone resorption, morphologic characterization of aortic plaques, plasma TMAO/TMA levels and plasma Choline/Creatinine/Betaine/L-Carnitine levels. **(A)** hemi-mandible from each group, as reconstructed by the micro-CT. **(B)** Cemento-enamel junction (red line: CEJ)-alveolar bone crest (green line: ABC) distance to represent alveolar bone loss (yellow line). **(C)** Representative Oil-Red-O stained aortic roots from control group and periodontitis group. **(D)** aortic root lesion area quantified using Oil-Red-O staining (original magnification of 400×). **(E)** representative fields of aortic root sections in control group stained with Hematoxylin–eosin. **(F)** representative fields of aortic root sections in experimental periodontitis group stained with Hematoxylin–eosin. **(G)** TMAO and TMA levels in plasma. **(H)** Choline, Creatinine, Betaine, and L-Carnitine levels in plasma. Data are presented as mean± SEM. (n = 5/group). *P < 0.05; P < 0.01.

Eight week old male ApoE^−/−^ mice were randomly divided into control and experimental periodontitis groups upon CMC and *P. gingivalis* administration. After 2 months of administration, the mandible specimens of the two groups were collected and scanned by Micro-CT. As shown in **Figure 1A**, the alveolar bone loss refers to the distance from the cementum-enamel junction (CEJ) to the alveolar crest (ABC) of the first molar. The results showed that the alveolar bone height of the periodontitis group was significantly lower than that of the control, and the difference was statistically significant (P <0.05) (**Figure 1B**). The experimental periodontitis group exhibited more plaque deposition under the intima than the control group (**Figure 1C**). Analysis of the atherosclerotic plaque area revealed a significant increase in the periodontitis group versus the control group (**Figure 1D**). The H&E-stained aortic sections were used for vessel wall assessment and invading mononuclear cell observation. As shown in **Figures 1E, F**, the experimental periodontitis group exhibited the lager intimal thickness, more plaque deposition under intima and more invading monocytes throughout arterial layers than those of the control group. As shown in **Supplemental Figure 1**, experimental periodontitis had significant effects on the serum lipid profile. The cholesterol levels shifted toward atherogenic levels.

The effect of experimental periodontitis on plasma TMAO, the important indicator of atherosclerosis, and its precursors were analyzed. The plasma TMAO level increased significantly in the experimental periodontitis group, compared to the control group. However, no significant difference was observed in the level of TMA and its precursors between the two groups, except that the plasma choline level decreased in the periodontitis group (P <0.01) (**Figures 1G, H**).

### Experimental Periodontitis Led to Changes in the Composition and Abundance of Intestinal Microflora

**Figure 2:**
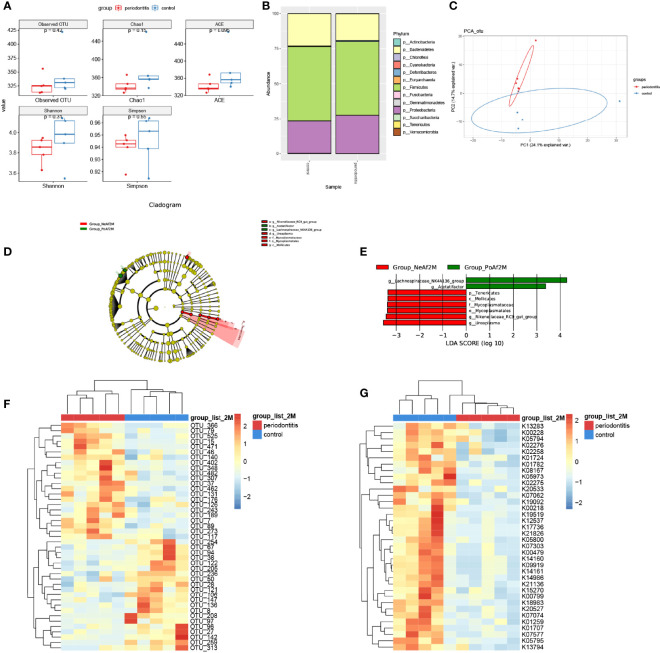
Gut microbiota alternation in periodontitis mouse model. **(A)** boxplot showed the value of the indicated index of alpha diversity of the periodontitis group and control group after 2-month treatment of *P. gingivalis*. **(B)** stack bar plot, showed the composition of microbiota at phylum level. **(C)** PCA plot of microbial composition. **(D)** linear discriminant analysis effect size (LEfSe)-based cladogram of fecal samples. **(E)** LDA scores of fecal samples. **(F)** Heatmap of sequenced bacterial operational taxonomic unit (OTU) abundances, scaled by row. **(G)** Heatmap of KEGG KO ORTHOLOGY analyzed by Tax4Fun2, scaled by row. 2M = 2 months after administration. The taxon name is preceded by one of the following: p_, phylum; f_, family; g_, genus; s_, species. Data are presented as mean ± SEM. (n = 5/group).

TMA, the precursor of TMAO, is a unique metabolite produced by the gut bacteria. The composition and abundance of intestinal microflora in the samples were detected by genome sequencing. There was no significant difference in the number of OTUs. The Chao1 index, ACE index, Shannon index, and Simpson index were lower in the periodontitis group than in the control group after a 2-month treatment (**Figure 2A**).

At the phylum level, the major 3 phyla of periodontitis and control mice were *Bacteroidetes*, *Firmicutes*, and *Proteobacteria*, and with no significant difference between the two groups (**Figure 2B**). Furthermore, the PCA analysis showed differences in microbiome composition between periodontitis and control mice (**Figure 2C**). The LEfSe analysis identified the characteristic bacteria at 2-month point. *Lachnospiraceae_NK4A136_group* and *Acetatifactor* were abundant in the periodontitis group, whereas *Rikenellaceae_RC9_gut_group* and *Mycoplasmataceae* were abundant in the control group (**Figures 2D, E**).

The analysis of different bacterial operational taxonomic units (OTUs) showed that 43 OTUs, including *Lachnospiraceae_bacterium_A4* (OTU402), *Lactobacillus_animalis* (OTU79), and *Parabacteroides_goldsteinii* (OTU147) at species level, and also *Lachnospiraceae_NK4A136_group* (OTU15, OTU471), *Mucispirillum* (OTU37), and *Ruminococcaceae_UCG-014* (OTU136), etc. at genus level, were significantly different between periodontitis model and control mice (**Figure 2F**). The full list of the OTU taxonomy is in **Supplemental Table 2**. As shown in **Figure 2G**, 37 KO ORTHOLOGY were at different levels between periodontitis and control mice, namely, K00108: choline dehydrogenase [EC:1.1.99.1], K00218: protochlorophyllide reductase [EC:1.3.1.33], K00228: coproporphyrinogen III oxidase [EC:1.3.3.3], etc.

### Experimental Periodontitis Led to the Increased Expression of the TMA Oxidase FMO3 in the Liver, Accompanied by Abnormal Intestinal Integrity, Liver Inflammation, and Enhanced LPS in the Peripheral Blood

**Figure 3:**
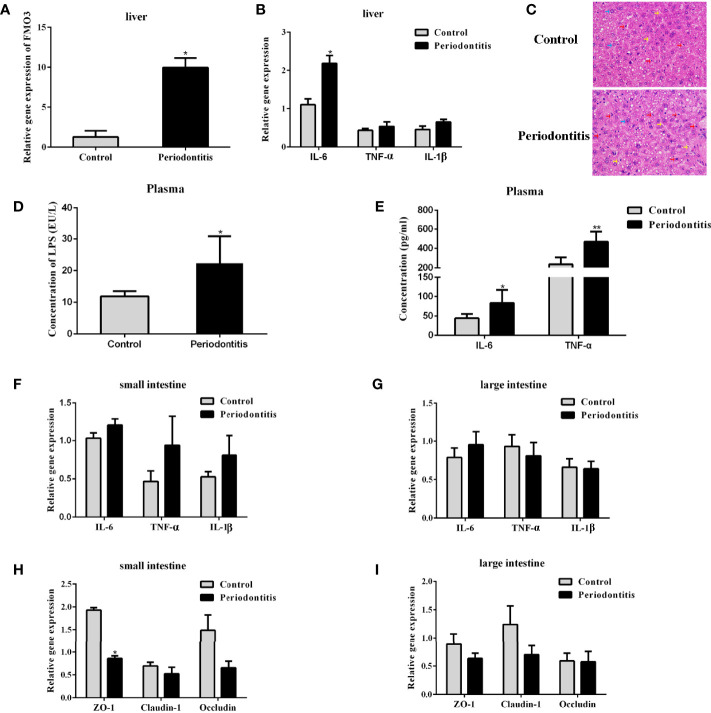
Comparisons of lipopolysaccharide and inflammatory factor concentrations in the plasma, liver, and intestine and the expressions of FMO3 and tight junction protein between the control group and the periodontitis group. **(A)** relative gene expression levels of FMO3 in in the liver tissue. **(B)** Relative gene expression levels of inflammatory cytokines in the liver tissue. **(C)** Effects of experimental periodontitis on histopathological changes of liver hepatocytes stained with H&E (original magnification of 400×). Blue arrows: hepatic cells. Red arrows: lymphocytes. Yellow arrows: sinusoids between the plates of hepatocytes. Black arrows: fat vacuoles. **(D)** concentration of lipopolysaccharide in the plasma. **(E)** Concentration of inflammatory cytokines in the plasma. **(F, G)** relative gene expression levels of inflammatory cytokines in the small intestine and large intestine. **(H, I)** Comparisons of relative tight junction gene expression levels in the small intestine and large intestine. Data are presented as mean ± SEM. (n = 5/group). *P <0.05; P < 0.01.

FMO3 is another important factor for the formation of TMAO. After the 2-month *P. gingivalis* administration, the mRNA expression of FMO3 was found to be significantly increased in the mouse livers (**Figure 3A**). Meanwhile, the expression of IL-6 (**Figure 3B**) in the liver of the periodontitis group was also enhanced. Pathological observation showed an increased lymphocyte content in the liver of the experimental periodontitis mice, compared with the control group. Experimental periodontitis also induced more steatosis, and the loss of cellular boundaries in livers (**Figure 3C**). Further blood analysis revealed that the experimental periodontitis mice had elevated LPS levels (P <0.05), while changes in inflammatory factors IL-6 and TNF-α were consistent with LPS (**Figures 3D, E**).

The mRNA expression of IL-6, TNF-α, and IL-1β in the small and large intestines did not differ significantly between the two groups (**Figures 3F, G**). The expressions of tight junction proteins ZO-1, Claudin-1, and Occludin were analyzed for gut barrier function. In the small intestine, experimental periodontitis significantly downregulated ZO-1 expression (P <0.05), and Claudin-1 and Occludin also showed less expression in experimental periodontitis mice (**Figure 3H**). These three tight junction proteins had similar decreased mRNA expressions in the large intestine at 8 weeks in the experimental periodontitis group (**Figure 3I**). The abnormal functions of intestinal immunity may create conditions for gut microbiota and its toxins like LPS to get into the bloodstream in mice.

### LPS-Induced Inflammation in the Liver Resulted in Enhanced FMO3 Expression and Plasma TMAO Level

**Figure 4:**
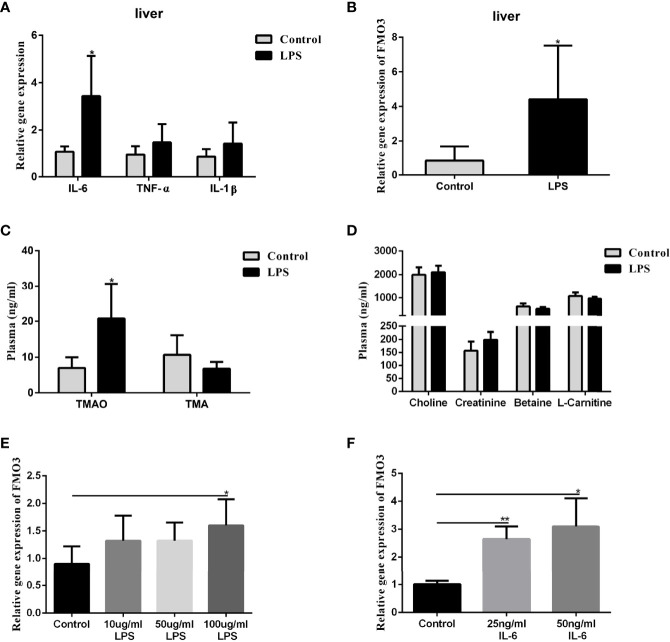
Effect of LPS on plasma TMAO/TMA levels, plasma Choline/Creatinine/Betaine/L-Carnitine levels, and the expression of FMO3 and inflammatory factors in the liver. Effect of LPS and IL-6 stimulation on FMO3 expression in HepG2 cell. **(A)** Relative gene expression levels of inflammatory cytokines in the liver tissue. **(B)** Relative gene expression levels of FMO3 in the liver tissue. **(C)** TMAO and TMA levels in plasma. **(D)** Choline, Creatinine, Betaine, and L-Carnitine levels in plasma. **(E)** comparisons of relative gene expression levels of FMO3 in the 10, 50, and 100 μg/ml LPS-treatment HepG2 cell. **(F)** relative gene expression levels of FMO3 in the 25, 50 ng/ml IL-6-treatment HepG2 cell. Data are presented as mean ± SEM. (n = 5/group). *P <0.05; P < 0.01.

To understand the effect of LPS incitation on the FMO3 in the liver and the oxidation of TMA, 8-week-old male C57BL/6J mice were injected with LPS intraperitoneally at a dose of 5 mg/kg every 3 days for 2 weeks. The level of the inflammatory factors IL-6, IL-1β, and TNF-α in the liver increased significantly, compared to the control (**Figure 4A**). The mRNA expression of FMO3 in the liver was also increased in the LPS group (**Figure 4B**). Meanwhile, the plasma TMAO significantly increased in the LPS group compared to that in the control group, while there was no obvious effect on the level of plasma TMA and its precursors (**Figures 4C, D**).

### Incitation of LPS or IL-6 to the HepG2 Cell Increased FMO3 Expression

The HepG2 cells were used to explore the direct effect of LPS and the immune effect of IL-6 on the expression of FMO3. The HepG2 cells were treated with 10, 50, and 100 μg/ml LPS, respectively, for 24 h. The mRNA expression of FMO3 increased significantly in the three LPS treatment groups, especially in the 100 μg/ml LPS group (P <0.05) (**Figure 4E**). Furthermore, a much more obvious increase of FMO3 expression was found after the treatment of HepG2 cells with 25 or 50 ng/ml IL-6 (**Figure 4F**).

## Discussion

In this study, we successfully established the periodontitis mouse model by oral administration of *P. gingivalis* and aggravated atherosclerosis. These results are consistent with our previous study (Yang et al., 2017), which affirmed the connection between periodontitis and general changes.

The most interesting finding was the increase of TMAO in the peripheral blood of the periodontitis mice. TMAO has been proven to be associated with cardiovascular risks by promoting inflammatory mediators, endothelial cell adhesion and foam cell formation, and decreasing reverse cholesterol efflux (Tang and Hazen, 2014; Chistiakov et al., 2015). TMAO is an oxidization product of TMA in the liver. TMA is a special metabolite of the gut microbiota. The intestinal microbiota synthesizes TMA from the choline in the diet. TMA is transported into the liver *via* the portal vein and then oxidized to TMAO by the catalytic action of FMOs. Diet, intestinal microbiota, and FMO3 are the primary factors affecting TMAO metabolism.

However, altered gut microbiota was observed in our study. When experimental periodontitis mice were compared to control mice, changes in the composition and abundance of intestinal microflora were found. The ACE index, Chao1 index, and Shannon index analysis showed that the community richness and diversity of gut microflora decreased after 2-month *P. gingivalis* administration, although the result was not statistically significant. *P. gingivalis* has shown antacid activity and may migrate to the colon and alter colon function (Sato et al., 2017). We speculated that experimental periodontitis might disturb the intestinal microflora, which would then interfere with TMA and TMAO production. Significant changes in gut bacteria, such as the *Lachnospiraceae-NK4A136_group* and *Bacteroidales_S24-7_group*, have been reported to be relevant to lipid metabolism and TMAO level (Wang et al., 2015; Skennerton et al., 2016), which may be indirect factors resulting in more serious lipid metabolism, atherosclerosis abnormalities and gut dysbiosis in experimental periodontitis mice. The microbiota and its function did change to an extent, although relationship between gut microbiota and the elevated TMAO was not very clear. The direct evidence of the relationship between the gut microbiota and TMAO needs further study.

We found that the expression of FMO3 in the liver was significantly increased and the livers of the experimental periodontitis mice were found in an inflammatory state. FMOs have been shown to regulate plasma TMAO levels effectively, and among the five members of the FMO family, FMO3 exhibits the highest specific activity towards TMA (Schugar and Brown, 2015). Our research hypothesized that this catalytic enzyme and the inflammatory response of the liver may contribute significantly to the progression of periodontitis, thereby promoting the development of atherosclerosis. The integrity disruption and inflammation of the intestine may give the channel to the bacteria and their toxins such as LPS. Furthermore, we verified the effect of IL-6 and LPS on the expression of FMO3 in the liver, and in HepG2 cells and observed the effect on the plasma TMAO level *in vivo*. It is an interesting finding that the experimental periodontitis could cause the inflammation state of the liver, and increase the FMOs. However, it still needs further research to identify. That is what we are going to focus on.

## Conclusion

Our research found that experimental periodontitis in ApoE^−/−^ mice induced gut dysbiosis, liver inflammation, and the increase in oxidase FMO3 and the intestinal metabolite TMAO. TMAO may play a key role in the development of atherosclerosis. These results suggest a possible mechanism that periodontitis induce atherosclerosis by influencing the intestinal microbes and the enterohepatic metabolism. It is possible to ameliorate the abnormality of TMAO metabolism through periodontal treatment in the future.

## Data Availability Statement

The data presented in the study are deposited in the SRA repository, accession number PRJNA784030.

## Ethics Statement

The animal study was reviewed and approved by the Institutional Animal Care and Use Committee of Nanjing University (IACUC-D2102033).

## Author Contributions

All authors have made substantial contributions to the conception and design of this study. SX, and QG contributed to conception and design of the research. LX and LH performed the experiments and wrote the paper. YW, DZ, and XZ fed the animals, and collected data. HM and SS implemented the information analysis. WS and QH were involved in the critical discussion of the research and revising it. All authors contributed to the article and approved the submitted version.

## Funding

This work was supported by the National Natural Science Foundation of China (81801041), Beijing, The Project of Invigorating Health Care through Science, Technology and Education (Grant No QNRC2016119) (2016–2020), Nanjing and the National Natural Science Foundation of China (82001111).

## Conflict of Interest

The authors declare that the research was conducted in the absence of any commercial or financial relationships that could be construed as a potential conflict of interest.

## Publisher’s Note

All claims expressed in this article are solely those of the authors and do not necessarily represent those of their affiliated organizations, or those of the publisher, the editors and the reviewers. Any product that may be evaluated in this article, or claim that may be made by its manufacturer, is not guaranteed or endorsed by the publisher.
