# Corrigendum: Experimental Periodontitis Deteriorated Atherosclerosis Associated With Trimethylamine N-Oxide Metabolism in Mice

**DOI:** 10.3389/fcimb.2022.919013

**Published:** 2022-06-15

**Authors:** Lingling Xiao, Lingyan Huang, Xin Zhou, Dan Zhao, Yan Wang, Haiyan Min, Shiyu Song, Weibin Sun, Qian Gao, Qingang Hu, Sijing Xie

**Affiliations:** ^1^Nanjing Stomatological Hospital, Medical School of Nanjing University, Nanjing, China; ^2^Center for Translational Medicine and Jiangsu Key Laboratory of Molecular Medicine, Medical School of Nanjing University, Nanjing, China; ^3^Department of Stomatology, The Second People’s Hospital of Taizhou, Taizhou, China; ^4^The Affiliated Stomatological Hospital of Soochow University, Suzhou, China; ^5^The Second Affiliated Hospital of Nanjing University of Chinese Medicine, Nanjing, China

**Keywords:** trimethylamine N-oxide, periodontitis, *Porphyromonas gingivalis*, gut microbiota, flavin-containing, monooxygenase 3

In the published article, there was an error in the affiliations superscripts assigned to some authors. “Lingling Xiao^1,2†^, Lingyan Huang^1†^, Xin Zhou^3^, Dan Zhao^1^, Yan Wang^1^, Haiyan Min^4^, Shiyu Song^5^, Weibin Sun^1^, Qian Gao^5*^, Qingang Hu^1*^ and Sijing Xie^1*^”.

The correct affiliation numbers for these authors should be: “Lingling Xiao^1,3†^, Lingyan Huang^1†^, Xin Zhou^4^, Dan Zhao^1^, Yan Wang^1^, Haiyan Min^5^, Shiyu Song^2^, Weibin Sun^1^, Qian Gao^2*^, Qingang Hu^1*^ and Sijing Xie^1*^”. The order and affiliation numbers remain unchanged

The authors apologize for this error and state that this does not change the scientific conclusions of the article in any way. The original article has been updated.

## Publisher’s Note

All claims expressed in this article are solely those of the authors and do not necessarily represent those of their affiliated organizations, or those of the publisher, the editors and the reviewers. Any product that may be evaluated in this article, or claim that may be made by its manufacturer, is not guaranteed or endorsed by the publisher.

