# Supplementary figures and images for: Experimental Periodontitis Deteriorated Atherosclerosis Associated With Trimethylamine N-Oxide Metabolism in Mice
Source: Front Cell Infect Microbiol. 2022 Jan 18;11:820535. doi: 10.3389/fcimb.2021.820535 (PMC8804528; doi:10.3389/fcimb.2021.820535)

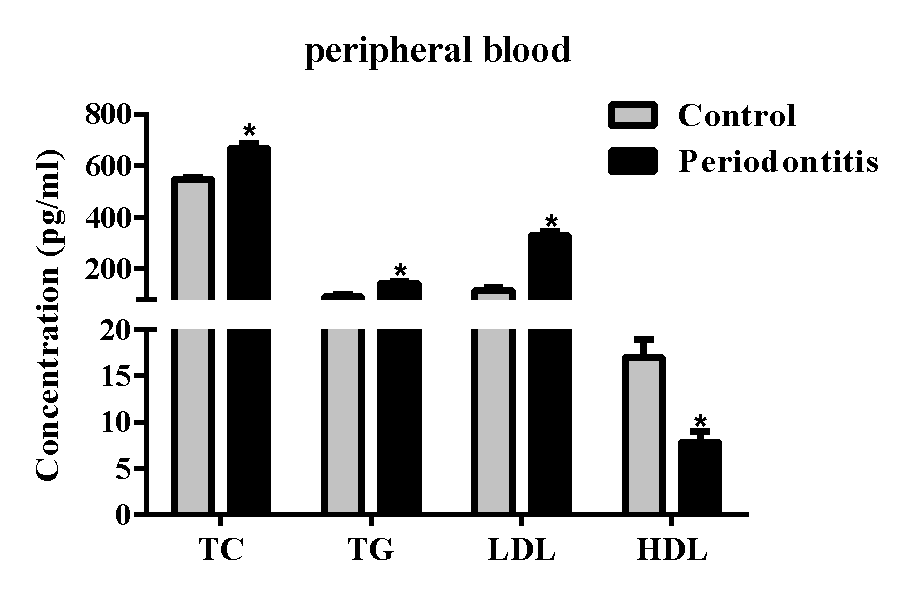

Supplement: Supplementary file 1 [file Image_1.tiff]
