# Supplementary material for: Experimental Periodontitis Deteriorated Atherosclerosis Associated With Trimethylamine N-Oxide Metabolism in Mice
Source: Front Cell Infect Microbiol. 2022 Jan 18;11:820535. doi: 10.3389/fcimb.2021.820535 (PMC8804528; doi:10.3389/fcimb.2021.820535)
Supplement: Supplementary file 2 [file Table_1.docx]

**Supplemental Table. 1. Primer sequence used in Quantitative Real-time PCR**

| Gene | Forward primer | Reverse primer |
| --- | --- | --- |
| *ZO-1* | *GATCCCTGTAAGTCACCCAGA* | *CTCCCTGCTTGCACTCCTATC* |
| *Claudin-1* | *GGGGACAACATCGTGACCG* | *AGGAGTCGAAGACTTTGCACT* |
| *Occludin* | *TTGAAAGTCCACCTCCTTACAGA* | *CCGGATAAAAAGAGTACGCTGG* |
| *IL-6* | *ACAAGTCGGAGGCTTAATTACACAT* | *TTGCCATTGCACAACTCTTTTC* |
| *TNF-α* | *TGGGACAGTGACCTGGACTGT* | *TCGGAAAGCCCATTTGAGT* |
| *IL-1β* | *TCGCTCAGGGTCACAAGAAA* | *CATCAGAGGCAAGGAGGAAAAC* |
| *FMO3* | *ACTTTGCCTTCTGTAAACGACATGA* | *GAACTTTACTGACGACACGCGTCT* |
| *GAPDH* | *ACCCCAGTTTACTCCATCCC* | *TGTTCCGGGTGGTTCTGCAG* |
| *Human-FMO3* | *GGGTCTGGGACAATGGTTATC* | *CAGAGATGGCTGTCGGTAAAT* |
| *Human-GAPDH* | *GGTGTGAACCATGAGAAGTATGA* | *GAGTCCTTCCACGATACCAAAG* |
