# Supplementary material for: Experimental Periodontitis Deteriorated Atherosclerosis Associated With Trimethylamine N-Oxide Metabolism in Mice
Source: Front Cell Infect Microbiol. 2022 Jan 18;11:820535. doi: 10.3389/fcimb.2021.820535 (PMC8804528; doi:10.3389/fcimb.2021.820535)
Supplement: Supplementary file 3 [file Table_2.docx]

**Supplemental Table. 2. The full list of the OTU taxonomy**

| OTU | Bacteria |
| --- | --- |

| OTU_7 | *k__Bacteria;p__Firmicutes;c__Clostridia;o__Clostridiales;f__Lachnospiraceae;g__;s__* |
| --- | --- |
| OTU_15 | *k__Bacteria;p__Firmicutes;c__Clostridia;o__Clostridiales;f__Lachnospiraceae;g__Lachnospiraceae_NK4A136_group;s__* |
| OTU_471 | *k__Bacteria;p__Firmicutes;c__Clostridia;o__Clostridiales;f__Lachnospiraceae;g__Lachnospiraceae_NK4A136_group;s__* |
| OTU_37 | *k__Bacteria;p__Deferribacteres;c__unidentified_Deferribacteres;o__Deferribacterales;f__Deferribacteraceae;g__Mucispirillum* |
| OTU_94 | *k__Bacteria;p__Bacteroidetes;c__Bacteroidia;o__Bacteroidales;f__Bacteroidales_S24-7_group;g__;s__* |
| OTU_38 | *k__Bacteria;p__Bacteroidetes;c__Bacteroidia;o__Bacteroidales;f__Bacteroidales_S24-7_group;g__;s__* |
| OTU_136 | *k__Bacteria;p__Firmicutes;c__Clostridia;o__Clostridiales;f__Ruminococcaceae;g__Ruminococcaceae_UCG-014;s__* |
| OTU_525 | *k__Bacteria;p__Firmicutes;c__Clostridia;o__Clostridiales;f__Lachnospiraceae* |
| OTU_259 | *k__Bacteria;p__Firmicutes;c__Clostridia;o__Clostridiales;f__Ruminococcaceae;g__Anaerotruncus;s__* |
| OTU_366 | *k__Bacteria;p__Firmicutes;c__Clostridia;o__Clostridiales;f__Lachnospiraceae* |
| OTU_402 | *k__Bacteria;p__Firmicutes;c__Clostridia;o__Clostridiales;f__Lachnospiraceae;g__Lachnospiraceae_NK4A136_group;s__Lachnospiraceae_bacterium_A4* |
| OTU_348 | *k__Bacteria;p__Firmicutes;c__Clostridia;o__Clostridiales;f__Lachnospiraceae;g__Lachnospiraceae_NK4A136_group;s__* |
| OTU_208 | *k__Bacteria;p__Cyanobacteria;c__Melainabacteria;o__Gastranaerophilales;f__;g__;s__* |
| OTU_462 | *k__Bacteria;p__Firmicutes;c__Clostridia;o__Clostridiales;f__Lachnospiraceae;g__;s__* |
| OTU_122 | *k__Bacteria;p__Bacteroidetes;c__Bacteroidia;o__Bacteroidales;f__Rikenellaceae;g__Rikenellaceae_RC9_gut_group;s__* |
| OTU_27 | *k__Bacteria;p__Bacteroidetes;c__Bacteroidia;o__Bacteroidales;f__Rikenellaceae;g__Rikenellaceae_RC9_gut_group;s__* |
| OTU_142 | *k__Bacteria;p__Tenericutes;c__Mollicutes;o__Mycoplasmatales;f__Mycoplasmataceae;g__Ureaplasma;s__* |
| OTU_79 | *k__Bacteria;p__Firmicutes;c__Bacilli;o__Lactobacillales;f__Lactobacillaceae;g__Lactobacillus;s__Lactobacillus_animalis* |
| OTU_147 | *k__Bacteria;p__Bacteroidetes;c__Bacteroidia;o__Bacteroidales;f__Porphyromonadaceae;g__Parabacteroides;s__Parabacteroides_goldsteinii* |
| OTU_46 | *k__Bacteria;p__Firmicutes;c__Clostridia;o__Clostridiales;f__Lachnospiraceae* |
| OTU_28 | *k__Bacteria;p__Bacteroidetes;c__Bacteroidia;o__Bacteroidales;f__Bacteroidales_S24-7_group;g__;s__* |
| OTU_106 | *k__Bacteria;p__Firmicutes;c__Clostridia;o__Clostridiales;f__Lachnospiraceae* |
| OTU_140 | *k__Bacteria;p__Proteobacteria;c__Deltaproteobacteria;o__Desulfovibrionales;f__Desulfovibrionaceae;g__Desulfovibrio* |
| OTU_236 | *k__Bacteria;p__Firmicutes;c__Clostridia;o__Clostridiales;f__Clostridiales_vadinBB60_group;g__;s__* |
| OTU_273 | *k__Bacteria;p__Firmicutes;c__Clostridia;o__Clostridiales;f__Clostridiales_vadinBB60_group;g__;s__* |
| OTU_8 | *k__Bacteria;p__Firmicutes;c__Clostridia;o__Clostridiales;f__Ruminococcaceae;g__;s__* |
| OTU_50 | *k__Bacteria;p__Bacteroidetes;c__Bacteroidia;o__Bacteroidales;f__Bacteroidaceae;g__Bacteroides* |
| OTU_482 | *k__Bacteria;p__Deferribacteres;c__unidentified_Deferribacteres;o__Deferribacterales;f__Deferribacteraceae;g__Mucispirillum;s__Mucispirillum_sp._69* |
| OTU_89 | *k__Bacteria;p__Firmicutes;c__Clostridia;o__Clostridiales;f__Lachnospiraceae* |
| OTU_205 | *k__Bacteria;p__Firmicutes;c__Clostridia;o__Clostridiales;f__Lachnospiraceae;g__;s__* |
| OTU_243 | *k__Bacteria;p__Firmicutes;c__Clostridia;o__Clostridiales;f__Ruminococcaceae;g__unidentified_Ruminococcaceae;s__[Clostridium]_leptum* |
| OTU_131 | *k__Bacteria;p__Firmicutes;c__Clostridia;o__Clostridiales;f__Lachnospiraceae;g__Acetatifactor;s__* |
| OTU_307 | *k__Bacteria;p__Deferribacteres;c__unidentified_Deferribacteres;o__Deferribacterales;f__Deferribacteraceae;g__Mucispirillum;s__* |
| OTU_117 | *k__Bacteria;p__Proteobacteria;c__Deltaproteobacteria;o__Desulfovibrionales;f__Desulfovibrionaceae;g__Bilophila;s__* |
| OTU_121 | *k__Bacteria;p__Bacteroidetes;c__Bacteroidia;o__Bacteroidales;f__Bacteroidales_S24-7_group;g__;s__* |
| OTU_97 | *k__Bacteria;p__Firmicutes;c__Clostridia;o__Clostridiales;f__Lachnospiraceae* |
| OTU_189 | *k__Bacteria;p__Proteobacteria;c__Alphaproteobacteria;o__Rhodospirillales;f__Rhodospirillaceae;g__Thalassospira;s__* |
| OTU_254 | *k__Bacteria;p__Firmicutes;c__Clostridia;o__Clostridiales;f__Lachnospiraceae* |
| OTU_126 | *k__Bacteria;p__Firmicutes;c__Clostridia;o__Clostridiales;f__Ruminococcaceae;g__Anaerotruncus;s__* |
| OTU_313 | *k__Bacteria;p__Firmicutes;c__Clostridia;o__Clostridiales;f__Ruminococcaceae;g__Ruminococcaceae_UCG-010;s__* |
| OTU_96 | *k__Bacteria;p__Bacteroidetes;c__Bacteroidia;o__Bacteroidales;f__Bacteroidales_S24-7_group;g__;s__* |
| OTU_67 | *k__Bacteria;p__Bacteroidetes;c__Bacteroidia;o__Bacteroidales;f__Bacteroidales_S24-7_group;g__;s__* |
| OTU_176 | *k__Bacteria;p__Firmicutes;c__Clostridia;o__Clostridiales;f__Ruminococcaceae;g__Ruminococcaceae_UCG-014;s__* |
